# Different modulation effects of 1 Hz and 20 Hz transcutaneous auricular vagus nerve stimulation on the functional connectivity of the periaqueductal gray in patients with migraine

**DOI:** 10.1186/s12967-021-03024-9

**Published:** 2021-08-17

**Authors:** Jin Cao, Yue Zhang, Hui Li, Zhaoxian Yan, Xian Liu, Xiaoyan Hou, Weicui Chen, Sierra Hodges, Jian Kong, Bo Liu

**Affiliations:** 1 Department of Psychiatry, Massachusetts General Hospital, Harvard Medical School, Charlestown, MA USA; 2 Department of Radiology, The Second Affiliated Hospital of Guangzhou University of Chinese Medicine, Guangzhou, Guangdong China; 3 Department of Neurology, The Second Affiliated Hospital of Guangzhou University of Chinese Medicine, Guangzhou, Guangdong China

**Keywords:** Transcutaneous auricular vagus nerve stimulation, Functional connectivity, Periaqueductal gray, Descending pain modulation network, Frequency

## Abstract

**Background:**

A growing body of evidence suggests that transcutaneous auricular vagus nerve stimulation (taVNS) may relieve symptoms of migraineurs. Frequency is one of the key stimulation parameters. The aim of this study is to investigate the modulation effect of taVNS frequency on the descending pain modulation system (DPMS) in patients with migraine.

**Methods:**

Twenty-four episodic migraineurs without aura (21 females) were recruited for the single-blind, crossover, functional magnetic resonance imaging (fMRI) study. Each participant attended two separate fMRI scan sessions, one for 1 Hz and another for 20 Hz taVNS, in a random order. Seed-based functional connectivity analysis was applied using the ventrolateral periaqueductal gray (PAG) as the region of interest.

**Results:**

Compared with the pre-taVNS resting state, continuous 1 Hz taVNS (during) produced a significant increase in functional connectivity between the PAG and the bilateral middle cingulate cortex (MCC), right precuneus, left middle frontal gyrus (MFG), and left cuneus. Compared with 20 Hz taVNS, 1 Hz taVNS produced greater PAG connectivity increases with the MCC, right precuneus/posterior cingulate cortex, left insula, and anterior cingulate cortex (ACC). A significant negative correlation was observed between the number of migraine attacks in the previous 4 weeks and the PAG-MCC functional connectivity in the pre-taVNS resting-state before 1 Hz taVNS.

**Conclusions:**

Our findings suggest that taVNS with different frequencies may produce different modulation effects on the descending pain modulation system, demonstrating the important role of stimulation frequency in taVNS treatment.

**Supplementary Information:**

The online version contains supplementary material available at 10.1186/s12967-021-03024-9.

## Background

The vagus nerve consists of a complex system that may regulate pain, mood, and the neuro-endocrine-immune axis [1–7]. Thus, stimulating the vagus nerve to modulate the function of the nerve and related organs has drawn the attention of clinicians and investigators for a long time. Anatomical studies found peripheral branches of the vagus nerve distributed on the ear [8, 9], and according to the bottom-up mechanism of the central nervous system, the propagation of electrical stimuli may follow an afferent path from the peripheral nerves towards the brain stem and central structures [10, 11]. Thus, direct stimulation of the nerve fibers on the ear may produce an effect similar to classic vagus nerve stimulation. This plausibility has led to the development of transcutaneous auricular vagus nerve stimulation (taVNS), a non-invasive, low-cost, and easily implementable alternative to classic vagus nerve stimulation [12–15]. A growing body of evidence suggests that taVNS can induce antinociception, which may affect peripheral and central nociception, inflammatory responses, autonomic activity, and pain-related behavior [1, 16–18].

While taVNS has demonstrated its potentials, the optimal parameters for taVNS, such as frequency, remain unclear [12]. Accumulating evidence suggests different frequencies may be associated with different physiological and treatment effects. For instance, investigators compared the effect of 2, 10, and 20 Hz stimulation on heart rate in healthy subjects, and they found that both 10 and 20 Hz could decrease heart rate [19]. Furthermore, studies suggest that the optimal taVNS frequency may vary across different disorders. For example, a recent clinical research study on taVNS treatment of drug-resistant epilepsy showed a significant reduction in seizure frequency in patients of the 25 Hz group compared to the 1 Hz group [20]. However, in another clinical study of migraine patients, investigators found that although both 1 Hz and 25 Hz taVNS improved clinical outcomes in patients with chronic migraine, 1 Hz taVNS produced greater improvement [21]. Nevertheless, the underlying mechanism of different taVNS frequencies remains unclear.

Recently, brain imaging has been widely used to investigate the central mechanism of taVNS, and these studies demonstrate that intermittent taVNS can modulate activity of certain brain regions consistent with the vagus nerve central projections [22–28]. For instance, investigators have assessed brainstem fMRI response to 2, 10, 25, and 100 Hz taVNS in healthy individuals, and found that the strongest brainstem response was evoked by 100 Hz stimulation [29]. In recent studies, we also applied the resting-state functional connectivity method to investigate the functional connectivity alteration during “the continuous taVNS” (20 Hz) and found that taVNS can modulate the functional connectivity of the ventral striatum and hypothalamus [30, 31].

Nevertheless, the neural substrates underlying frequency have rarely been investigated in a patient population such as migraine; elucidating how different frequencies can modulate pathways associated with migraine may further facilitate the development of this promising neuromodulation method.

Recently, the role of descending pain modulatory system (DPMS) in pain modulation and the physiopathology of chronic pain has drawn more and more attention [32–34]. Yet, investigating the functional status of the DPMS in humans remains a challenge. In an earlier study [35], we investigated the resting state functional connectivity (rsFC) of the periaqueductal grey (PAG), a key region in the DPMS in healthy subjects and found significant rsFC between the PAG and central regions of the DPMS, such as the anterior cingulate cortex (ACC), rostroventral medulla (RVM) and anterior insula, demonstrating the feasibility of using functional connectivity methods to non-invasively investigate the DPMS in humans.

Following the study, the PAG functional connectivity has been applied to investigate the physiopathology of chronic pain disorders including migraine [35–43], menstrual pain [44–46], postherpetic neuralgia [47], fibromyalgia [48], myofascial pain [49], visceral pain [50], low back pain [36], and neck pain [51]. Further, studies have also shown that effective treatment can significantly modulate the PAG functional connectivity in patients with migraine [41], chronic low back pain [52], and knee osteoarthritis [53]. We also found that continuous electroacupuncture stimulation alters PAG functional connectivity [54]. Taken together, these findings demonstrate the important role of PAG functional connectivity in pain research.

Thus, in this study, we investigate how continuous taVNS at 1 Hz versus 20 Hz (a relatively low frequency versus a moderate frequency) that are widely applied in taVNS studies [12] can modulate the PAG functional connectivity in patients with migraine without aura, using a cross-over design. We hypothesize that taVNS at 1 Hz versus 20 Hz may produce greater PAG functional connectivity changes due to its greater improvement in patients with migraine [21].

## Methods

The study was conducted in accordance with the Declaration of Helsinki, and the protocol was approved by the Ethics Committee of the Second Affiliated Hospital of Guangzhou University of Chinese Medicine (Z2016-079-01). All participants provided written informed consent before starting the study.

### Participants

Twenty-four episodic migraineurs without aura were recruited in the present study from outpatient neurology clinics of the Second Affiliated Hospital of Guangzhou University of Chinese Medicine. Similar to our previous studies [55, 56], the diagnosis of migraine was based on the International Classification of Headache Disorders, 2nd Edition (ICHD-II), as diagnosed by a specialist working at the neurology outpatient service.

Patients were eligible for participation if they: (1) were 18 to 45 years of age, (2) self-reported being right-handed, (3) have at least 6 months of migraine duration, (4) have at least one headache attack per month, (5) have not taken any prophylactic headache medications during the past 4 weeks, (6) have not taken any psychoactive or vasoactive drugs during the past 3 months. Patients were excluded if there was a/an: (1) headache induced by other diseases, (2) headache attack within 48 h prior to the experiment or during the experiment, (3) pregnant or lactating, (4) any other chronic pain conditions, (5) severe head deformity or intracranial lesions, (6) score on the Self-Rating Depression Scale [57] or Self-Rating Anxiety Scale [58] > 50, and (7) inability to provide informed consent for oneself.

### Study design

**Fig. 1:**
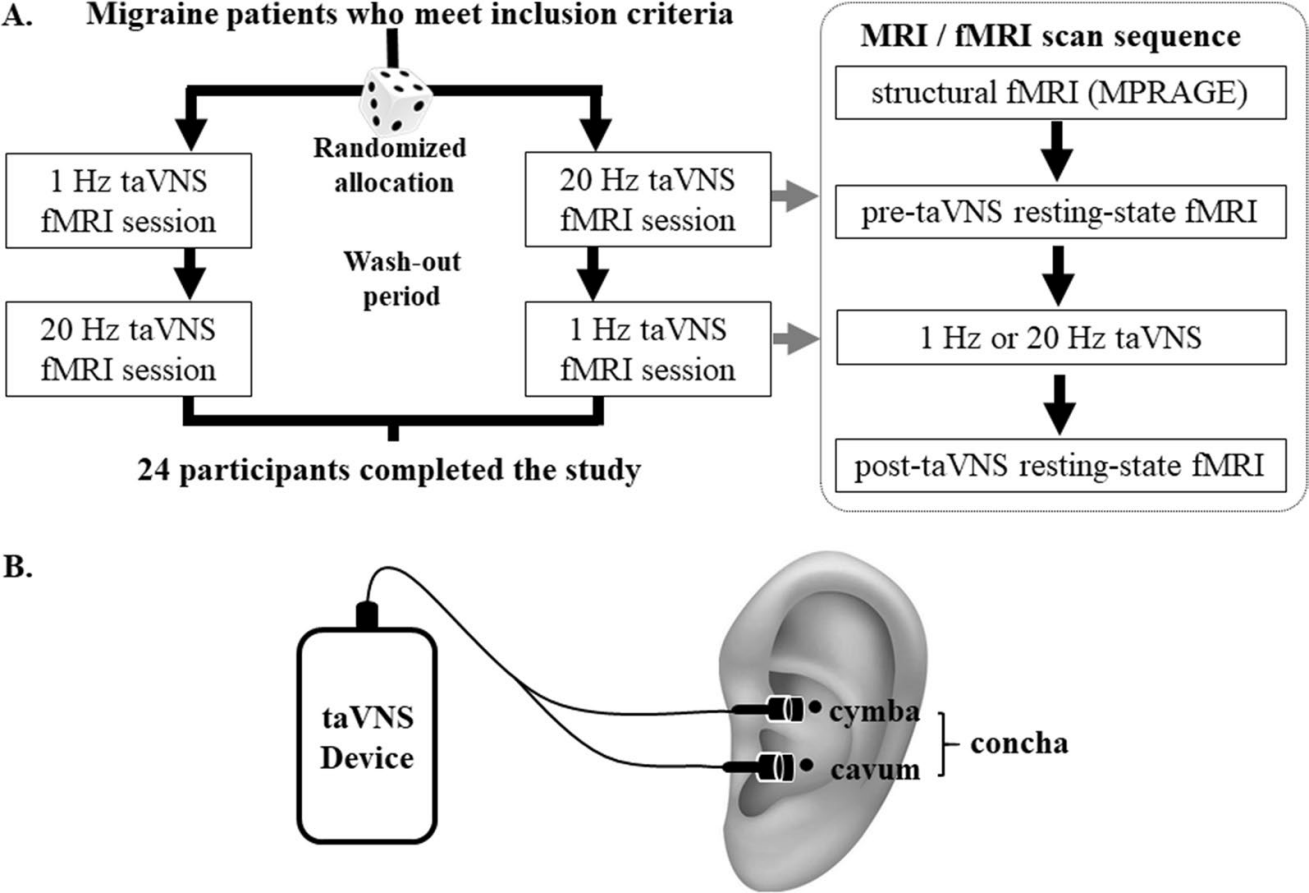
Overview of study procedure and taVNS sites. **A** Study procedure. **B** taVNS sites

A single-blind, crossover functional magnetic resonance imaging (fMRI) trial design was applied in the present study to investigate the modulation effects of 1 Hz and 20 Hz taVNS in patients with migraine without aura. Specifically, each participant attended two taVNS fMRI scan sessions with identical parameters, one for 1 Hz and another for 20 Hz taVNS in a random order (Fig. 1A). Each session was separated by at least 7 days to avoid sensitization to the stimuli. All scans were applied during an interictal period when the participants were free from headache symptoms.

### Interventions

In the current study, we applied taVNS on the participant’s left concha (cymba and cavum, Fig. 1B, Additional file 1: Fig. S1) [59]. The electrical stimulation was performed using the Electronic Acupuncture Treatment Instrument (SDZ IIB, Huatuo, Suzhou, China) with the self-made MRI compatible electrode to deliver electric current at 1 Hz or 20 Hz with a continuous wave (width: ~ 0.2 ms). The 1 Hz/20 Hz taVNS stimulation lasted about 8 min. Similar to our previous studies, stimulation current intensity was adjusted to the strongest nonpainful sensation that participants could tolerate (approximately 4 mA) [31, 55, 60–62].

### Clinical assessments

Migraine duration, migraine attacks during the past 4 weeks, and average migraine intensity of the past 4 weeks on the 0 (“not at all”) to 100 (“extremely”) visual analog scale (VAS) were assessed preceding the first MRI scan session. Participants were also asked to complete the Migraine Specific Quality-of Life Questionnaire [63] to measure the impact of migraine on health‐related quality of life.

### MRI data acquisition

All imaging data was acquired at the Second Affiliated Hospital of Guangzhou University of Chinese Medicine using a 3 T MRI System (Siemens MAGNETOM Verio 3.0 T, Erlangen, Germany) with a 24-channel phased-array head coil. Each scan session included a pre-taVNS resting-state fMRI (8 min), the 1 Hz or 20 Hz continuous taVNS (8 min) fMRI, and a post-taVNS resting-state fMRI (8 min).

fMRI scans were acquired with the following parameters: time repetition = 2000 ms, time echo = 30 ms, flip angle = 90°, field of view = 224 mm × 224 mm, matrix size = 64 × 64, slice thickness = 3.5 mm with 0.7 mm inter-slice gap, 31 axial slices paralleled and 240 time points. During the fMRI scans in resting-state and continuous taVNS, participants were asked to stay awake, keep their heads still, eyes closed, and ears plugged and to not think about any particular thing. A T1-weighted structural image was acquired by an isotropic multi-echo magnetization-prepared rapid gradient-echo pulse sequence for anatomic localization of significant signal changes: time repetition = 1900 ms, time echo = 2.27 ms, flip angle = 9°, field of view = 256 mm × 256 mm, data matrix = 256 × 256, and slice thickness = 1.0 mm.

### Functional connectivity analysis

Data and calculations of functional connectivity were conducted using the CONN toolbox version 18.b (http://www.nitrc.org/projects/conn) [64]. We used the default preprocessing pipeline for seed-to-voxel functional connectivity analysis. The specific steps were as follows: functional realignment and unwarping, slice timing correction, head motion correction, co-registration of the anatomical image to the mean functional image, segmentation of the anatomical gray matter, white matter, and CSF, normalization to Montreal Neurological Institute (MNI) 152 standard template and smoothing with a 6-mm full width at half maximum (FWHM) kernel. A default frequency window of 0.008 to 0.09 Hz was used for band-pass filtering.

To eliminate correlations caused by head motion and artifacts, we identified outlier time points in the motion parameters and global signal intensity using ART (http://www.nitrc.org/projects/artifact_detect). Images whose composite movement exceeded 0.5 mm or whose global mean intensity was greater than three standard deviations from the mean image intensity were treated as outliers. The time series of the head motion matrix of outliers was also entered as first-level covariates.

Similar to our previous studies [35, 36, 41], we selected the right ventrolateral periaqueductal gray (vlPAG) with a 2 mm radius sphere (MNI coordinates x = 4, y = − 26, z = − 14) as the region of interest (ROI). In addition, we also chose seeds with a 2 mm radius in the fourth ventricle (MNI coordinates: x = 4, y = 10, z = 12; x = − 4, y = 10, z = 12) as a control. Seeds were created using the SPM Wake Forest University Pickatlas toolbox (http://fmri.wfubmc.edu/software/pickatlas) [65].

In the first-level analysis, we produced a correlation map for each participant by extracting the blood oxygenation level dependent time course separately from the vlPAG and the control seeds and computing Pearson’s correlation coefficients between the time course in the vlPAG/control seeds and every voxel of the whole brain. Correlation coefficients were Fisher transformed into “z” scores to increase normality.

In seed-to-voxel functional connectivity analyses, we first used a pairwise t-test to compare the vlPAG-based functional connectivity between the pre-taVNS resting-state and during continuous taVNS (1 Hz and 20 Hz taVNS, respectively). Next, we compared the difference of vlPAG-based functional connectivity change (during continuous taVNS minus pre-taVNS resting-state) between 1 and 20 Hz taVNS. Finally, we compared the vlPAG-based functional connectivity difference between the pre-taVNS and post-taVNS resting-state between the 1 Hz and 20 Hz taVNS.

For whole brain analysis, a threshold of voxel-wise p < 0.005, and p_FDR_ < 0.05 at cluster level was applied. Also, given the important role of the anterior cingulate cortex (ACC), medial prefrontal cortex (mPFC), insula, amygdala, and thalamus in the DPMS [32, 36, 53, 66] and pathophysiology of migraine [41, 67–72], we pre-defined these areas as regions of interest (ROIs), and derived masks of each region from the Automated Anatomical Labeling brain atlas using the Wake Forest University Pickatlas toolbox as ROIs. A threshold of voxel-wise p < 0.005 was used in data analysis. Similar to previous studies [73–75], Monte Carlo simulations using the 3dFWHMx and 3dClustSim (as part of the Analysis of Functional NeuroImages program [http://afni.nimh.nih.gov] released in July 2017) were applied for the p value correction for pre-defined ROIs. For each region, the minimum voxel size required for p < 0.05 cluster level p value correction is indicated as the k value in the results presented below.

To explore the association between the initial clinical assessments and the vlPAG-based pre-taVNS resting-state functional connectivity for 1 Hz and 20 Hz respectively, we also selected significantly altered vlPAG-based connectivity clusters (during continuous taVNS minus pre-taVNS resting-state) and extracted the average z-score values of peak MNI of clusters above significance in vlPAG-based pre-taVNS resting-state. Correlation analyses were conducted using the R program in JASP open-source statistical software (Version 0.8.1, http://www.jasp-stats.org), and p values were Bonferroni corrected (see “Results” for details).

## Results

### Demographic and clinical assessments

Twenty-four participants completed the study and were included in the data analysis [21 females; age 31.33 ± 1.55 years, mean ± standard error (SE)]. No participant reported administration of acute migraine medication or having an attack 48 h prior to the MRI sessions. Detailed results for demographic and clinical assessments are shown in Table 1. All participants reported acceptable stimulation intensity underneath the electrodes during the continuous taVNS, with no adverse effects reported. The interval period of the two taVNS/fMRI scan sessions was 8.79 ± 0.74 (mean ± SE) days.

**Table 1:** Demographic and clinical assessments

Demographic	
Participant count	24
Sex (female/male)	21/ 3
Age (mean ± SE, yrs)	31.33 ± 1.55
Clinical assessments	
Migraine duration (mean ± SE, yrs)	8.68 ± 1.47
Migraine attacks (mean ± SE)	1.67 ± 0.25 (ranging from 1 to 5)
VAS (mean ± SE)	38.60 ± 3.30
MSQ (mean ± SE)	74.83 ± 1.90
SDS (mean ± SE)	42.14 ± 1.82
SAS (mean ± SE)	39.69 ± 1.85

Migraine attacks assessed attack times during the past 4 weeks. The VAS assessed the average migraine intensity of the 4 weeks preceding the first MRI scan. The MSQ, SDS, SAS were assessed preceding the first MRI scan

*VAS* visual analog scale, *MSQ* Migraine Specific Quality of Life, *SDS* Self-rating Depression Scale, *SAS* Self-rating Anxiety Scale

### vlPAG-based functional connectivity analysis results

**Fig. 2:**
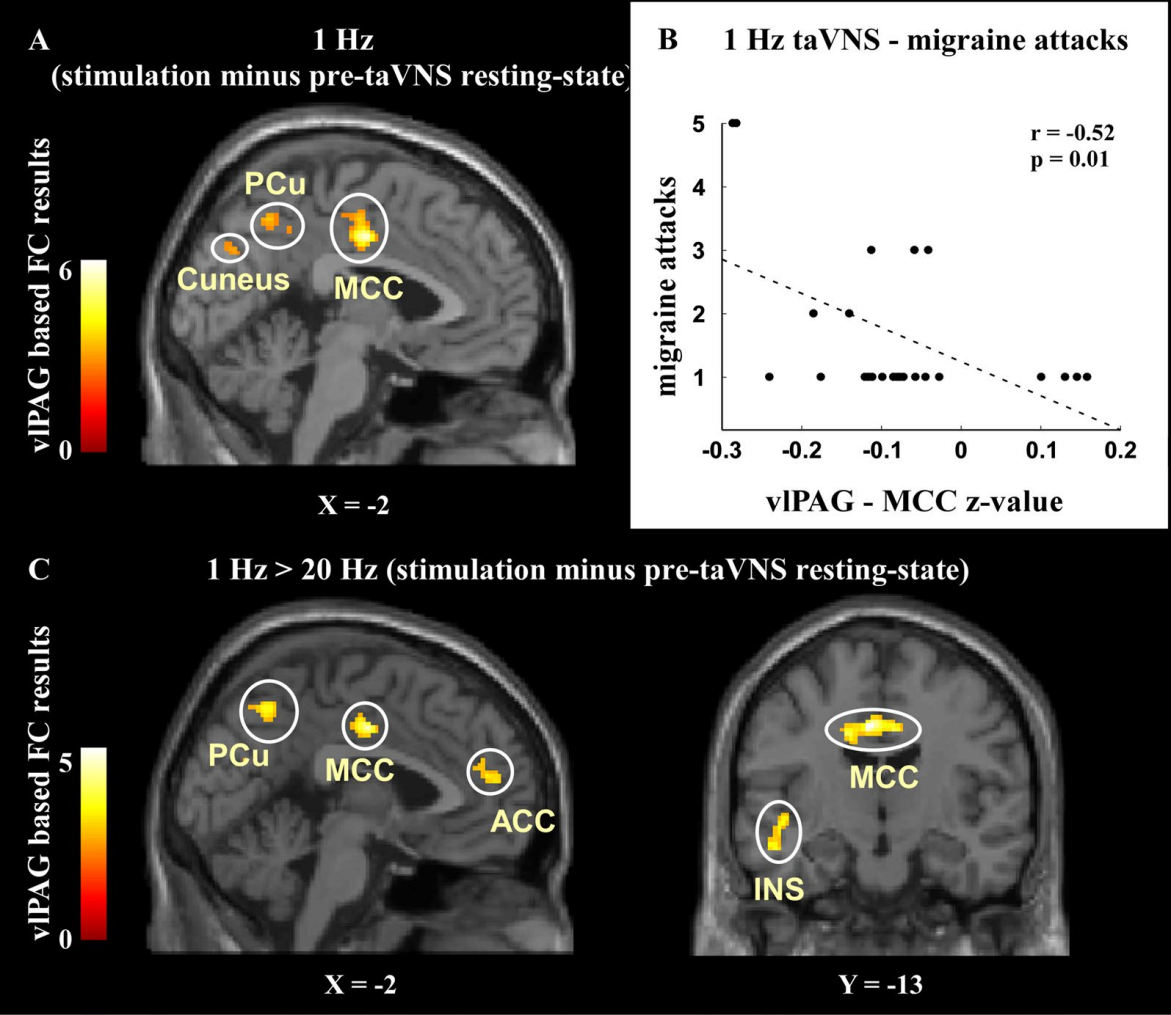
vlPAG based connectivity results. **A** Compared to pre-taVNS resting-state, 1 Hz taVNS had significantly greater connectivity with the MCC, precuneus, Cuneus, and left MFG (not present in the figure). **B** Significant negative correlation was observed in migraine attacks and vlPAG-MCC connectivity in pre-taVNS resting-state preceding to the 1 Hz taVNS. Bonferroni correction was applied, and the significance threshold was adjusted to p < 0.0125 because four significant clusters were identified. **C** Compared to 20 Hz, 1 Hz taVNS had significant connectivity increases (stimulation minus pre-taVNS resting-state) with the MCC, precuneus, ACC, and left insula. *FC* functional connectivity, *vlPAG* ventrolateral periaqueductal gray, *MCC* middle cingulate cortex, *PCu* precuneus, *MFG* middle frontal gyrus, *ACC* anterior cingulate cortex, *INS* insula

Compared with pre-taVNS resting state, 1 Hz continuous taVNS (during) produced significant functional connectivity increases between the vlPAG and the bilateral middle cingulate cortex (MCC), the right precuneus, the left middle frontal gyrus (MFG), and the left cuneus (Table 2, Fig. 2A). There was no significant finding detected when we applied the same analysis on the 20 Hz taVNS data set.

**Table 2:** Comparisons of the vlPAG functional connectivity change in 1 Hz and 20 Hz taVNS

Comparisons	Brain Regions	Cluster size (voxel number)	Peak T	MNI coordinates		
				x	y	z
1 Hz taVNS > pre-resting	MCC	282	6.36	− 4	− 12	40
	PCu	200	4.71	4	− 50	44
	MFG	232	4.56	− 30	30	42
	Cuneus	191	4.05	− 6	− 76	32
pre-resting > 1 Hz taVNS						
20 Hz taVNS > pre-resting	No regions survive the threshold					
pre-resting > 20 Hz taVNS						

“Pre-resting” indicated pre-taVNS resting-state. Results were significant at cluster p_FDR_ < 0.05, corrected at the whole brain level

*vlPAG* ventrolateral periaqueductal gray, *MCC* middle cingulate cortex, *PCu* precuneus, *MFG* middle frontal gyrus

In addition, we compared the vlPAG-based connectivity difference in 1 Hz vs. 20 Hz taVNS [(during 1 Hz taVNS minus 1 Hz pre-taVNS resting-state) vs. (during 20 Hz taVNS minus 20 Hz pre-taVNS resting-state)], and found that compared to 20 Hz, 1 Hz taVNS produced greater vlPAG-based connectivity increases with the MCC, the right precuneus/posterior cingulate cortex (PCC), the left insula (k = 18), and the anterior cingulate cortex (ACC) (k = 41) (Table 3, Fig. 2C). No significant decrease in vlPAG-based functional connectivity was detected.

**Table 3:** Comparisons of vlPAG functional connectivity change produced by 1 Hz and 20 Hz taVNS

Comparisons	Brain Regions	Cluster size	Peak T	MNI coordinates		
				x	y	z
1 Hz > 20 Hz	MCC	225	5.37	− 6	− 12	40
	PCu/PCC	216	4.74	4	− 48	46
	*INS	148	5.35	− 44	− 6	− 6
	*ACC	45	3.50	− 4	42	18
20 Hz > 1 Hz	No regions survive the threshold					

Change presented in continuous taVNS minus pre-taVNS resting-state. “*” identified results significant at cluster p < 0.05 after 3dFWHMx and 3dClustSim correction. Other results were significant at cluster p_FDR_ < 0.05 corrected at the whole brain level

*vlPAG* ventrolateral periaqueductal gray, *MCC* middle cingulate cortex, *PCu* precuneus, *PCC* posterior cingulate cortex, *INS* insula, *ACC* anterior cingulate cortex

With the threshold we set, no significant result has been found in the comparison of vlPAG-based pre- and post-taVNS resting-state functional connectivity differences between the 1 Hz and 20 Hz taVNS.

We found that 1 Hz taVNS increased vlPAG resting state functional connectivity with the MCC, precuneus, MFG and cuneus compared with the pre-taVNS resting state. To explore the potential clinical meaning of these functional connectivity increases, we performed correlation analyses between the vlPAG resting-state functional connectivity with these regions during the pre-taVNS (1 Hz) and the clinical measures (migraine attacks in the past 4 weeks and VAS). Results showed a significant negative correlation between the number of preceding migraine attacks and the vlPAG-MCC functional connectivity in the pre-taVNS resting-state preceding the 1 Hz taVNS (r = − 0.52, p = 0.01, significant after Bonferroni correction p < 0.05/4 = 0.0125 because four significant clusters were identified, please see Table 2 and Fig. 2B for details). No other significant vlPAG-based functional connectivity finding was detected.

We also performed the above analysis using bilateral seeds from the fourth ventricle. No result was found at the threshold we set in functional connectivity analysis.

## Discussion

In the present study, we compared the vlPAG connectivity changes evoked by 1 Hz and 20 Hz taVNS in migraine patients. Results showed that compared to pre-taVNS resting-state, continuous 1 Hz taVNS produced increased connectivity in the MCC, MFG, precuneus and cuneus. Compared to 20 Hz, 1 Hz taVNS produced greater connectivity increases in the MCC, ACC, precuneus and left insula. There is a significant negative association between migraine attacks in the past 4 weeks and the vlPAG-MCC connectivity during resting-state. Our findings suggest that taVNS with different stimulation frequencies may produce different modulation effects on the descending pain modulation system.

As a non-invasive and safe peripheral neuromodulation method, taVNS has been applied in a wide range of disorders such as depression, epilepsy, tinnitus, migraine, as well as cognitive and behavioral disorders [12, 21, 76–78]. Nevertheless, one challenge for the development of taVNS is to elucidate the modulation effect of taVNS with different parameters so that we can optimize its effects for different disorders.

As a key parameter of taNVS, frequency is a continuous measurement. Thus, it is not possible to test/compare the effects of different frequencies in one study. As a start of this line of work, we have chosen 1 Hz as a representative of low frequency. The 20 Hz frequency has been used to treat depression, and previous studies have found that 20 Hz taVNS can significantly modulate the multiple brain networks [27, 60–62], particularly the functional connectivity of the amygdala [62], default mode network [60], hypothalamus [79], and ventral striatum [30], all of which are associated with pathophysiology of migraine [80, 81]. Further, investigators found that 20 Hz taVNS in healthy subjects could decrease heart rate [19], and 20 Hz is also close to the higher frequency used in a previous study in which the authors have compared the treatment effect of 1 Hz and 25 Hz in [21]. Thus, we have chosen 20 Hz to represent a moderate frequency in this study.

We found that continuous 1 Hz taVNS can significantly increase vlPAG-MCC connectivity. In addition, the vlPAG-MCC connectivity during resting-state before 1 Hz taVNS was negatively associated with participants’ migraine attacks. Literature suggests that the MCC is involved in the affective, cognitive, attention, and orienting aspects of pain [82–84]. A previous study found that migraine is associated with decreased grey matter at the MCC [85] and increased activation during experimental heat pain (compared to healthy controls). The pain-induced MCC activation is associated with migraine attacks in migraineurs [86]. Interestingly, we found that the vlPAG-MCC connectivity increased during 1 Hz taVNS, but not during 20 Hz taVNS, which may provide a neural mechanistic support to a previous clinical trial [21], in which researchers investigated the therapeutic effects of daily 1 Hz and 25 Hz taVNS on chronic migraineurs over 3 months, and demonstrated that 1 Hz taVNS was more prominent in migraine alleviation.

Furthermore, we observed that continuous 1 Hz taVNS can produce vlPAG-rACC connectivity increases compared to 20 Hz. In addition, we also detected an increase in vlPAG-rACC connectivity (compared to resting-state) at a less conservative threshold (p = 0.01, cluster size = 14). The rACC is a key region of the DPMS [35, 36], and contains numerous opioid receptors [87]. Previous studies have suggested that the rACC plays an important role in the pathophysiology of migraine [88, 89]. Findings from the current study are consistent with our prior study, in which migraine patients are associated with reduced connectivity of the PAG-rACC, compared to healthy subjects, and effective acupuncture treatment can normalize the decreased connectivity in PAG-rACC correspondingly [41]. Further, the study demonstrates that a DPMS abnormality might be an underlying pathological mechanism of migraine, and such an abnormality can be normalized by effective treatment.

In addition, we found that 1 Hz taVNS can increase vlPAG connectivity with the precuneus and cuneus. The precuneus is a key region in the default mode network. Studies suggest that the default mode network (DMN) is a pivotal network affected by migraine [90–92]. We found that migraineurs showed decreased functional connectivity between the right frontoparietal network and precuneus compared with healthy controls, and the connectivity significantly increased after effective treatment [93]. In a more recent study, we found abnormal posterior thalamus (pulvinar nucleus) dynamic network functional connectivity with the precuneus, and the changes were significantly correlated with the headache frequency of migraine [79].

The cuneus is a key region of the visual network. In a recent longitudinal study on grey matter volume of migraineurs, researchers found that migraineurs developed a decreased grey matter volume of visual regions, including the cuneus. The decreased volume was associated with the level of migraine severity, in terms of disease duration, pain intensity, and attack frequency [94]. We found migraine is associated with altered posterior thalamus dynamic network functional connectivity with the visual cortex [79], and the abnormal functional connectivity within the visual, default mode, sensorimotor, and frontal-parietal networks, which could discriminate migraineurs from healthy controls, with 93% sensitivity and 89% specificity [95]. More recently, we found that 4-week taVNS at 1 Hz can decrease the connectivity between the occipital cortex-related thalamus subregion and the postcentral gyrus/precuneus [96]. Taken together, these studies demonstrate the important role of the precuneus and cuneus in the pathology of migraine. Our study further suggests that 1 Hz taVNS may modulate the connectivity between the descending pain modulation system, the default mode network, and the visual network.

Nevertheless, the question whether the effective frequency of taVNS that influences migraine is different from other diseases remains open [19, 20, 97]. Further studies are needed to determine the optimal frequency of taVNS for different diseases. Additionally, as a brain imaging study, the aim of this study was to investigate and compare if 1 Hz and 20 Hz taVNS can modulate the vlPAG functional connectivity in a migraine population rather than assessing the efficacy/clinical effects of 1 Hz and 20 Hz taVNS. In addition, we used seeds in the ventricle as a control ROI, and the lack of significant results further validated our findings.

Potential limitations of this work include a relatively small sample size of migraine participants with low-frequency migraine attacks. Future studies are needed to investigate if the findings can be replicated in migraineurs with high attack frequencies in a larger sample size. Also, there are only three male participants (of 24 in total) included in this study. This ratio is partly consistent with epidemiology studies showing the prevalence rate of female migraineurs is much higher than male migraineurs [98]. Nevertheless, we have applied a cross-over design, which should have controlled the potential gender effects in this study. This study is not designed to answer the question of gender differences. A future study is needed to elucidate if male and female migraineurs are associated with same taVNS response.

Furthermore, our MRI scans were applied when participants were migraine-free, so we could not assess the acute effects of taVNS on headache intensity. Also, clinical trials on migraine usually assess the clinical improvement (migraine attack time or pain intensity) in the past month, thus, we could not investigate/compare the clinical improvement produced by single 1 Hz/20 Hz taVNS treatment, as well as the association between functional connectivity changes (evoked by 1 Hz and 20 Hz taVNS) and clinical improvement. Moreover, although still under investigation, some studies suggest that different stimulation frequencies of taVNS may induce different changes in heart rate, which can be considered as a confounding factor of functional connectivity [19, 99]. Nevertheless, the heart rate changes evoked by taVNS are relatively small, and studies also show no significant change on blood pressure values after taVNS [100]. Future study should consider measuring this confounding factor and adjust for it during data analysis.

## Conclusion

In summary, we found continuous 1 Hz taVNS can significantly modulate functional connectivity between the vlPAG and key regions of the DPMS in patients with migraine. Our findings demonstrate the important role of stimulation parameters (particularly the frequency) in taVNS treatment of different disorders.

## Supplementary Information

**Additional file 1: Figure S1.** Electrodes and clips used in this study.

## Data Availability

Data supporting the findings of this study are available from the corresponding author, upon reasonable request.

## Abbreviations

taVNS: Transcutaneous auricular vagus nerve stimulation
DPMS: Descending pain modulation system
DMN: Default mode network
MRI: Magnetic resonance imaging
fMRI: Functional magnetic resonance imaging
PAG: Periaqueductal gray
vlPAG: Ventrolateral periaqueductal gray
ACC: Anterior cingulate cortex
rACC: Rostral anterior cingulate cortex
MCC: Middle cingulate cortex
PCC: Posterior cingulate cortex
MFG: Middle frontal gyrus
RVM: Rostroventral medulla
mPFC: Medial prefrontal cortex
ROI: Regions of interest
FWHM: Full width at half maximum
VAS: Visual analog scale
