# Supplementary material for: Different modulation effects of 1 Hz and 20 Hz transcutaneous auricular vagus nerve stimulation on the functional connectivity of the periaqueductal gray in patients with migraine
Source: J Transl Med. 2021 Aug 17;19:354. doi: 10.1186/s12967-021-03024-9 (PMC8371886; doi:10.1186/s12967-021-03024-9)
Supplement: Supplementary file 1 — Additional file 1: Figure S1. Electrodes and clips used in this study. [file 12967_2021_3024_MOESM1_ESM.docx]

**Additional file 1**

**Different modulation effects of 1 Hz and 20 Hz transcutaneous auricular vagus nerve stimulation on the functional connectivity of the periaqueductal gray in patients with migraine**

**
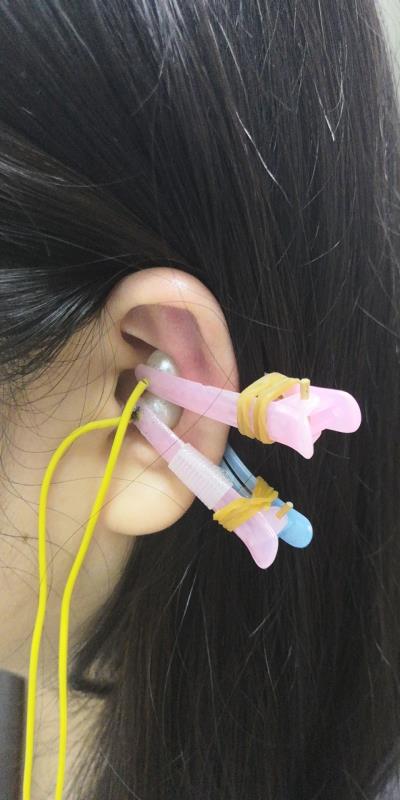
**

**Figure S1.** Electrodes and clips used in this study.
